# Address of Dr. W. W. Shyrock, President of the Indiana State Dental Association, at Indianapolis, June 30th, 1896

**Published:** 1896-08

**Authors:** 


### Address of Dr. W. W. Shryock, President of the Indiana State Dental Association, at Indianapolis, June 30, 1896.

*My Esteemed Fellows of the Indiana Dental Association:*

After extending a most cordial greeting, allow me to express my profound appreciation of the honor conferred in my election to the most honorable office within your gift, and ask that the generous favor which prompted it may continue in my behalf throughout our deliberations.

We meet to-day to plant the mile-post that marks our thirty-eighth birthday. On December 28, 1858, a few unselfish and noble spirits—men from the chief towns in the State—met at Indianapolis and perfected an organization which is a power and pride, commanding our mutual gratitude and congratulations.

Like all movements destined to great accomplishments, it had an humble beginning. Those who took this important initiatory step deserve a grateful remembrance, and their names can not be too often brought to our view. They were: Drs. John F. Johnson, T. H. Nichols, P. G. C. Hunt, G. C. North, A. M. Moore, J. P. Ulery, John Hood, S. B. Smith, H. R. Hurd, George Lupton, H. Salterect, A. W. French, S. B. Harland, W. R. Webster and D. C. Dills.

Few survive to receive our benediction, but their works live after them. Since our last meeting another one from this honored roll has been taken from us. It is a just tribute to say that Dr. P. G. C. Hunt, for fifty years, stood without a peer in his profession in Indiana. In acknowledment of which this Association, and the profession at large, will ever be found ready to honor and revere his memory.

In our commendable eagerness to add to our membership, in a regretful way we often ask: 'Why are so few, compared to the many, seeking the benefits and lending their influence to our organized efforts?" It is a demonstrated fact, and the time has come when we must recognize an immutable natural law, which is operating for our greatest good in withholding from our Association an unwieldy and diversely disposed assemblage. It is error to assume that the members now in attendance at this Association, as in all previous ones, are not the acknowledged and willingly-accepted representatives of all the dentists of Indiana, and morally delegated to act in their interests with a principle more binding than if elected by ballot.

The truth of this position was demonstrated at the time of securing the State Dental Law. At that time the State contained five hundred dentists; of this number, not to exceed fifty expressed a desire for its passage, and less than half that number took any active part in advocating it.

Where were the silent four hundred and fifty? They were eloquent in verifying the adage that, "Silence gives consent." To be sure some antagonistic muttering and grumbling, the sweet prerogative of natural-born critics on all questions of progress, were heard from ambush, but moral cowardice restrained any open obstruction to a measure destined to give the greatest good to the largest number—a factor in the great law of human progress and civilization.

An enumeration of the membership of the American Dental Association shows only two hundred and seventeen. Embraced in this are the National Association of Dental Faculties, and the National Board of Dental Examiners, This Association dominates the destiny of the profession in America. It is, so to speak, a sovereign power which rules 25,000 subjects.

It can not be an arbitrary power, hence it must be by the consent of the governed. Assuming that this position is not a mere hypothesis, but conclusively evident, let us waste no more time in vain regret or depressing reflection, but accept the fact that we are Heaven-ordained to represent the silent members of our profession in Indiana; that they fully acquiesce, their consent being affirmed by their absence, in accepting us as the elect.

This ground does not in the least admit of any laxity in vigilance or diligence, but rather increases our ardor and activity in winning additional recruits to maintain their chosen quota of representation. This commission imposes upon us greater responsibilities and duties. Let us now enter upon them determined to discharge them conscientiously in accordance with our highest moral conception—"With charity for all and malice toward none."

